# Linking O-GlcNAc and intron retention

**DOI:** 10.7554/eLife.107226

**Published:** 2025-05-08

**Authors:** John A Hanover

**Affiliations:** 1 Cell Biochemistry Section at the National Institute of Diabetes and Digestive and Kidney Diseases, National Institutes of Health Bethesda United States

**Keywords:** intron retention, O-glcnac, OGT, gene regulation, SFSWAP, decoy exon, O-GlcNAcase, Human

## Abstract

An elegant screening strategy unveils a molecular actor that connects widespread changes in mRNA processing with a nutrient-sensing protein modification.

**Related research article** Govindan A, Conrad NK. 2025. SFSWAP is a negative regulator of OGT intron detention and global pre-mRNA splicing. *eLife* **13**:RP104439. doi: 10.7554/eLife.104439.

A key but often overlooked step in gene regulation is the processing of precursor messenger RNAs, the molecules that first emerge from the transcription of a gene. These pre-mRNAs contain sequences (exons) encoding information required to make a protein, interspersed with non-coding regions (introns) that must typically be removed before protein production can start. A large molecular machine, the spliceosome, distinguishes introns from exons, removes the former and joins the latter to create a mature mRNA template.

Cells can fine-tune these complex splicing events to control what proteins are made, when, and in what form (Boutz et al., 2015). Thus, splicing allows the organism to meet changing demands quickly and flexibly. Deliberately leaving in ‘detained introns’, for example, prevents target pre-mRNAs from being exported from the nucleus, which allows the cell to delay or prevent the production of certain proteins without degrading the associated RNA transcripts (Yap et al., 2012). Exon skipping, on the other hand, occurs when the spliceosome skips an exon to allow an mRNA to be produced albeit with an altered coding sequence. ‘Decoy’ exons also occur. Although their mode of action remains unclear, these exons contained within introns are believed to recruit and then ‘stall’ the spliceosome, preventing it from proceeding with the normal splicing process.

Recent work has shown that a highly dynamic protein modification, known as O-GlcNAc, plays a key role in regulating detained intron splicing (Tan et al., 2020). O-GlcNAcylation consists of the addition of a small sugar molecule (GlcNAc) onto certain amino acids, which can alter the activity and location of thousands of proteins in a cell. It helps modulate gene expression and many crucial signaling pathways, such as those involved in responding to DNA damage or maintaining cell identity in early development (Bond and Hanover, 2015; Zachara et al., 2022; Fehl and Hanover, 2022). O-GlcNAc levels vary in response to broader environmental signals, in particular stressors or variations in nutrient availability. As such, this process allows cells to adjust their response and retain their internal balance in the face of ever-changing conditions. Finely regulating O-GlcNAcylation is therefore crucial for survival, with deregulation being linked to some forms of X-linked intellectual disability (Konzman et al., 2020; Vaidyanathan et al., 2017).

Interestingly, the control of O-GlcNAcylation itself seems to be linked to detained intron splicing (Tan et al., 2020). Two enzymes, O-GlcNAc transferase (OGT) and O-GlcNAcase (OGA), respectively add and remove GlcNAc to/from proteins (Bond and Hanover, 2015; Zachara et al., 2022). An intricate feedback loop maintains stability in the system: when O-GlcNAc levels rise, for instance, OGT production decreases while that of OGA increases. More precisely, under high O-GlcNAc levels, introns are retained in the pre-mRNA transcript of the OGT gene, preventing protein expression. A similar mechanism unfolds for OGA when O-GlcNAc is low (Figure 1A and B; Park et al., 2017; Tan et al., 2020). Should this fail to restore healthy levels of O-GlcNAc, the cell responds by specifically changing detained intron splicing across the whole genome. What controls this O-GlcNAc-driven splicing regulation, however, is still poorly understood. Now, in eLife, Ashwin Govindan and Nicholas Conrad from the University of Texas Southwestern Medical Center report having identified the splicing factor SFSWAP as a key player required for this process (Figure 1C; Govindan and Conrad, 2025).

**Figure 1.**
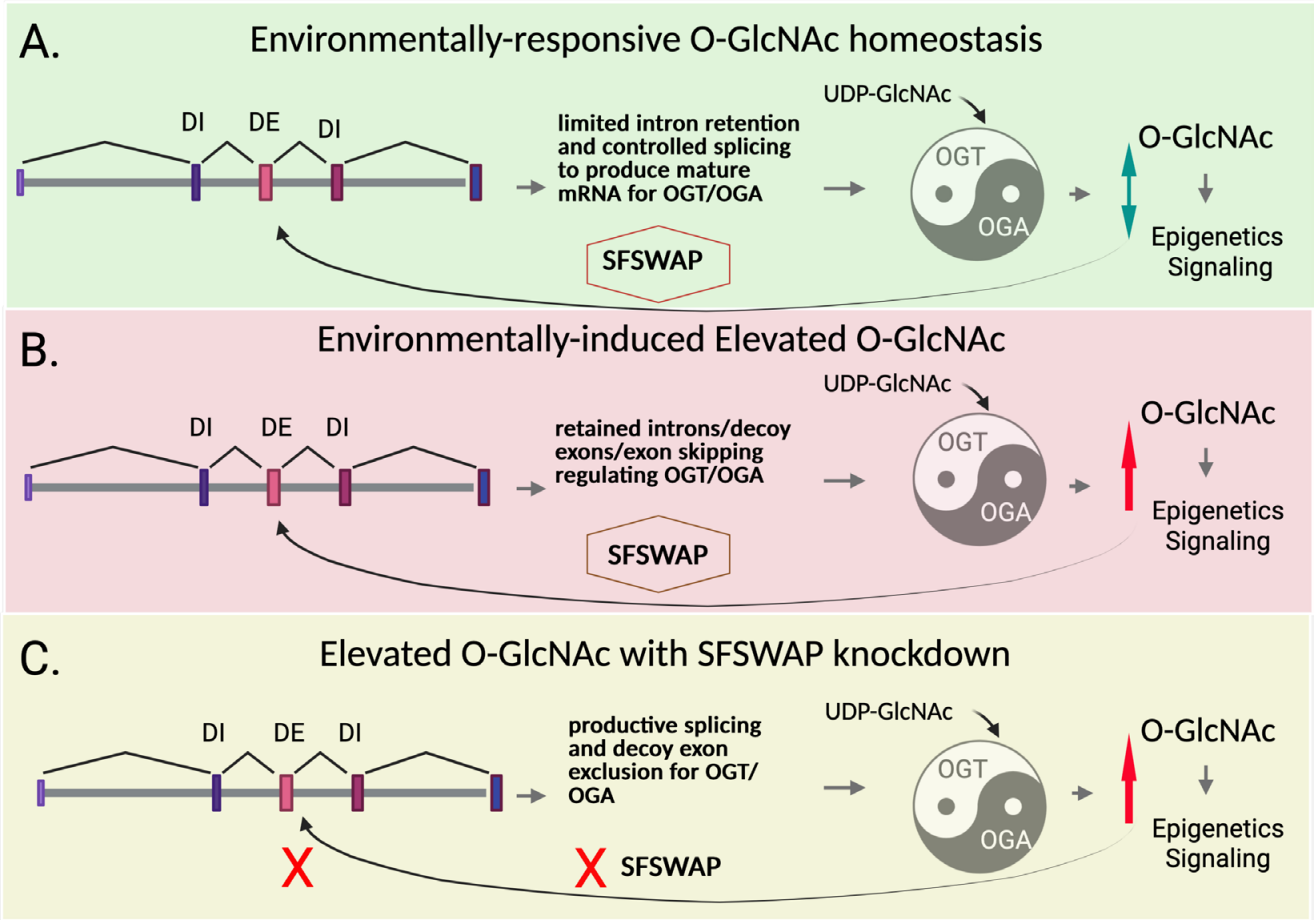
SFSWAP as a regulator of O-GlcNAc through splicing. O-GlcNAc is a protein modification that acts as an important regulator of epigenetics and signaling. The amount of O-GlcNAcylation within a cell changes in response to the environment, in particular nutrient availability, and it is subject to intense control. (**A**) O-GlcNAc balance (homeostasis; green arrow) is normally maintained by carefully adjusting the production of OGT and OGA, the enzymes that respectively increase and decrease O-GlcNAc levels (represented by a yin-yang symbol). O-GlcNAc levels (double green arrow) must be maintained for proper signaling and epigenetic regulation of gene expression (right). The levels of both OGT and OGA are maintained partially by regulation of detained intron splicing (although OGA is regulated in a similar way, only the relevant portion of the OGT gene is shown in the figure). This process involves altering how OGT transcripts are processed to produce mature mRNAs; in particular, how coding (grey) and non-coding sequences (various shades of purple and pink) are recognized, excised and joined by the cell machinery to create a useable template for protein synthesis. When detained intron (DI) splicing takes place, non-coding introns and decoy exons are retained in the transcript, preventing protein production. The work of Govindan and Conrad reveals that a splicing factor known as SFSWAP helps regulate this process under the influence of O-GlcNAc levels. (**B**) When O-GlcNAc is elevated (red arrow) due to nutrient or environmental factors, SFSWAP promotes intron retention (as well as exon skipping) in the OGT transcript; as a result, OGT production decreases, helping to re-establish balance in the synthesis of O-GlcNAc. A related mechanism regulates OGA splicing via intron retention. (**C**) Deleting SFSWAP while under high O-GlcNAc levels (red arrow) blunts this feedback mechanism (red crosses); more transcripts are produced in which introns have been removed, in part because cells failed to recognize decoy exons. A similar mechanism regulates OGA intron retention. These experiments also suggest that SFSWAP plays a more global role in the negative regulation of mRNA splicing and acts as an overall positive regulator of intron retention.

The team focused on the gene coding for OGT, devising an elegant screening strategy to identify the molecular actors that contribute to its splicing under O-GlcNAc control. They first created an OGT-based genetic construct that appropriately responded to O-GlcNAc levels, and whose detained intron splicing could easily be detected. This reporter was formed of the sequence coding for the fluorescent protein GFP, in which intron 4 from OGT was introduced (alongside the corresponding exons 4 and 5); if this intron was retained, GFP production dropped. Intron 4 was chosen in part because it contains a ‘decoy exon’, recruiting spliceosomes but blocking normal splicing (Parra et al., 2018).

Govindan and Conrad introduced their construct in human cell lines in which they systematically deleted genes one at a time. The cells were then manipulated so that their O-GlcNAc levels increased or decreased, and GFP expression was carefully monitored. A range of genes emerged as potentially controlling OGT splicing under these conditions, including several that code for components of the spliceosome. One particularly promising candidate was then selected based on rigorous criteria: SFSWAP, a splicing factor whose equivalent in *Drosophila* regulates alternative splicing in various transcripts, including its own. In humans, SFSWAP is known to control the splicing of several genes (including Tau, CD45 and fibronectin) by inhibiting the inclusion of specific exons.

Next, Govindan and Conrad investigated how SFSWAP contributed to five types of splicing events (detained introns, skipped exons, alternate 5´ splice sites, alternate 3´ splice sites and mutually exclusive exons) under various O-GlcNAc levels. Overall, the analyses show that SFSWAP acts on two of these mechanisms – detained introns and skipped exons – and that it promotes the retention of introns in a wide range of transcripts.

Additional experiments examining the impact of SFSWAP on pre-mRNAs with or without decoy exons led Govindan and Conrad to propose that this factor increases intron retention by acting at a later stage in the splicing cycle. According to this model, SFSWAP may interfere with how the spliceosome proceeds to excise certain sequences, but not how this machinery assembles at specific locations on the transcript. Taken together, these findings have important implications for understanding the complex interplay between environmentally responsive O-GlcNAc metabolism and the global regulation of splicing – uncovering a feedback loop by which global changes can directly influence gene expression at a post-transcriptional level.

## References

[bib1] Bond MR, Hanover JA (2015). A little sugar goes a long way: the cell biology of O-GlcNAc. The Journal of Cell Biology.

[bib2] Boutz PL, Bhutkar A, Sharp PA (2015). Detained introns are a novel, widespread class of post-transcriptionally spliced introns. Genes & Development.

[bib3] Fehl C, Hanover JA (2022). Tools, tactics and objectives to interrogate cellular roles of O-GlcNAc in disease. Nature Chemical Biology.

[bib4] Govindan A, Conrad NK (2025). SFSWAP is a negative regulator of OGT intron detention and global pre-mRNA splicing. eLife.

[bib5] Konzman D, Abramowitz LK, Steenackers A, Mukherjee MM, Na HJ, Hanover JA (2020). *O*-GlcNAc: regulator of signaling and epigenetics linked to X-linked intellectual disability. Frontiers in Genetics.

[bib6] Park SK, Zhou X, Pendleton KE, Hunter OV, Kohler JJ, O’Donnell KA, Conrad NK (2017). A conserved splicing silencer dynamically regulates O-GlcNAc transferase intron retention and O-GlcNAc homeostasis. Cell Reports.

[bib7] Parra M, Booth BW, Weiszmann R, Yee B, Yeo GW, Brown JB, Celniker SE, Conboy JG (2018). An important class of intron retention events in human erythroblasts is regulated by cryptic exons proposed to function as splicing decoys. RNA.

[bib8] Tan ZW, Fei G, Paulo JA, Bellaousov S, Martin SES, Duveau DY, Thomas CJ, Gygi SP, Boutz PL, Walker S (2020). O-GlcNAc regulates gene expression by controlling detained intron splicing. Nucleic Acids Research.

[bib9] Vaidyanathan K, Niranjan T, Selvan N, Teo CF, May M, Patel S, Weatherly B, Skinner C, Opitz J, Carey J, Viskochil D, Gecz J, Shaw M, Peng Y, Alexov E, Wang T, Schwartz C, Wells L (2017). Identification and characterization of a missense mutation in the O-linked β-N-acetylglucosamine (O-GlcNAc) transferase gene that segregates with X-linked intellectual disability. The Journal of Biological Chemistry.

[bib10] Yap K, Lim ZQ, Khandelia P, Friedman B, Makeyev EV (2012). Coordinated regulation of neuronal mRNA steady-state levels through developmentally controlled intron retention. Genes & Development.

[bib11] Zachara NE, Akimoto Y, Boyce M, Hart GW, Varki A, Cummings RD, Esko JD, Stanley P, Hart GW, Aebi M, Mohnen D, Kinoshita T, Packer NH, Prestegard JH, Schnaar RL, Seeberger PH (2022). Essentials of Glycobiology.

